# Recruitment of abundant NK cells to the PFAPA tonsils support the crucial role of innate immunity in pathogenesis of PFAPA syndrome

**DOI:** 10.1186/1546-0096-13-S1-O82

**Published:** 2015-09-28

**Authors:** S Chiesa, R Caorsi, I Ingrosso, F Bellora, F Penco, A Bertoni, C Pastorino, A Omenetti, M Finetti, S Borghini, A Sementa, R D'Agostino, A Martini, M Gattorno

**Affiliations:** 1 G.Gaslini Institute, Laboratory of Immunology and Rheumatic Diseases, UO Pediatria II, Genoa, Italy; 2 University of Genoa, Medicina Sperimentale, Genoa, Italy; 3 G.Gaslini Institute, Laboratoy of Molecular Genetics, Genoa, Italy; 4 G.Gaslini Institute, Anatomic Pathology, Genoa, Italy; 5 G.Gaslini Institute, UO Otorinolaringoiatria, Genoa, Italy

## Introduction

Periodic fever, aphthous stomatitis, pharyngitis, and cervical adenitis (PFAPA) syndrome is a more frequent cause of recurrent fever in children. The exact etiology of this pediatric disorder is still unknown. Palatine tonsils are sites where innate immunity leads to the onset of adaptive immunity, mediated by B and T lymphocytes.

## Objective

Our purpose is to understand if infiltrating inflammatory cells in PFAPA tonsils contribute to evolving disease.

## Methods

We have collected tonsil samples from 2 groups of pediatric patients undergoing tonsillectomy: PFAPA patients (n=20) and children who had indication of bacterial tonsillitis (control group, CG) (n=20). We have performed a precise phenotypic analysis of subpopulations on tonsil cells and tissues using flow cytometry and immunohistochemistry.

## Results

During the asymptomatic phase the number of monocytes did not differ between the PFAPA and control tonsils. We observed a considerable recruitment of NK cells in tonsils of PFAPA patients with respect to CG. Surprisingly, we have detected a significant expansion of both CD56^bright^CD16^-^ and CD56^dim^CD16^+^NK cell subsets when compared to CG. Strict characterization of activating and inhibitory NK receptors revealed a crucial role of CD56^dim^CD16^+^. Specially, activating receptors, such as natural cytotoxicity receptors (NCRs) and NKG2D, are higher in NK of PFAPA patients. Furthermore, FACS analysis displayed a higher number of naïve and a significantly lower percentage of effector memory CD4^+^ and CD8^+^ T cells in PFAPA patients compared to CG. Additionally, PFAPA patients presented a significant decrease of functional follicular helper T cells (Tfh) and regulatory T cells.

## Conclusions

These results confirm a relevant involvement of NK cells in pathogenesis of PFAPA supporting the crucial role of the innate immunity. Nonetheless, the abundant and activated NK cell subsets (particularly CD56^dim^CD16^+^) might influence adaptive immune responses demonstrated impaired in the tonsils of PFAPA patients.

